# Glycine Betaine Treatment Maintains Postharvest Quality of Hupingzao Jujube Fruit by Enhancing the Antioxidant System

**DOI:** 10.3390/foods14193385

**Published:** 2025-09-30

**Authors:** Fei Shi, Jinbin Wu, Zifan Geng, Yuqing Xing, Yulei Zhang, Zhigang Li, Tengfei Wang, Yu Wang

**Affiliations:** 1 College of Food Science and Engineering, Shanxi Agricultural University, Jinzhong 030801, China; shifei@sxau.edu.cn (F.S.); jinbin_wu0311@163.com (J.W.); 18334839499@163.com (Z.G.); x1184093485@163.com (Y.X.); zyl0101@126.com (Y.Z.); sxndlzg@163.com (Z.L.); wangtengfei@sxau.edu.cn (T.W.); 2 Shanxi Provincial Technology Innovation Center for Fruit and Vegetable Storage, Preservation and Processing, Jinzhong 030801, China

**Keywords:** Hupingzao jujube, refrigeration, fruit quality, membrane integrity, glycine betaine

## Abstract

Postharvest Hupingzao jujubes are prone to softening and reddening during storage. To investigate the influence of glycine betaine on the fruit quality of cold-stored jujubes, the jujubes were immersed in a 15 mmol L^−1^ glycine betaine solution for 10 min, then stored at 0 ± 1 °C for 100 days. The relevant physical and chemical quality indicators were determined every 20 days. The results indicated that glycine betaine treatment effectively maintained fruit firmness, reduced weight loss, slowed down the respiration rate, and lessened the redness of the fruit peel. Compared with the control group, the malondialdehyde and H_2_O_2_ contents in glycine betaine-treated fruit decreased by 26.65% and 9.04%, respectively. In addition, glycine betaine treatment elevated the contents of non-enzymatic antioxidants, including ascorbic acid, total flavonoids, total phenols, and proanthocyanidins. Meanwhile, the activities of superoxide dismutase, peroxidase, catalase, and ascorbate peroxidase in the jujube fruit were enhanced after glycine betaine treatment. In conclusion, glycine betaine treatment preserved the quality of the jujube fruits by enhancing the antioxidant metabolism. The results establish a scientific basis for the potential application of glycine betaine in postharvest fruit preservation, providing a strategy to mitigate storage-related quality deterioration.

## 1. Introduction

The Hupingzao jujube (*Ziziphus jujuba* Mill.) is produced in Jinzhong, Shanxi, China. It is usually harvested in the mid-early period of September at the white mature stage, when the peel is white-green and the flesh is hard [1,2]. After harvest, the jujube is easy to redden, soften, dehydrate, and shrink, negatively influencing the fruit quality and commodity price [3]. Hupingzao is deeply loved by consumers for its big size and high nutrient content when it is eaten fresh, but it is susceptible to a fast ripening and senescence after harvest when stored at room temperature [4,5,6]. Refrigeration is conventionally employed to extend the postharvest shelf life by inhibiting the ripening processes and suppressing microbial decay. However, prolonged refrigeration induces fruit pulp softening via alcoholic fermentation, accompanied by a progressive peel discoloration (red-brown pigmentation), leading to a significant degradation in both nutritional composition and organoleptic properties [7,8]. This quality deterioration has constrained the development of value-added jujube products and remains a critical challenge for the industry. Consequently, developing effective preservation technologies to maintain refrigerated jujube quality represents a research priority.

Glycine betaine (GB) is ubiquitous in plants and has an important role in enhancing stress resistance [9,10]. GB is a secondary product of metabolism and a very important osmotic adjustment substance; it maintains the cell membrane stability and integrity, protects protein structure, keeps enzymes stabilized, and improves cold tolerance during the postharvest cold storage of horticultural crops [11,12]. In recent years, GB treatment has been reported to alleviate postharvest chilling injuries in many higher plant products. For example, GB treatment modulated phenolic and sugar metabolisms and reduced the chilling injury of peach fruit [13]; regulated proline metabolism and antioxidant enzyme systems to inhibit the pericarp browning of “Nanguo” pear fruits [14]; and enhanced antioxidant defense systems and ameliorated chilling injury in banana and blood orange during cold storage [15,16]. In particular, GB treatment maintained the cell membrane integrity and cell wall strength of the winter jujube and inhibited the postharvest softening and quality decline of the jujube fruit [3]. These studies indicate that GB treatment may be an effective approach to maintain jujube fruit quality. However, there are obvious differences in the storage of the winter jujube and Hupingzao jujube. Although the skin of the Hupingzao is thicker and the moisture content is lower than that of the winter jujube, it is easier to soften and has a shorter storage time than the winter jujube. And the research about the role of GB treatment in alleviating the quality deterioration of the Hupingzao jujube fruit during cold storage is limited.

Therefore, we investigated the effect of GB treatment in preserving the quality of Hupingzao jujubes during refrigerated storage. The study systematically analyzed its impacts on key antioxidant bioactive compounds, including ascorbic acid, total flavonoids, total phenolics, and proanthocyanidins. Furthermore, we elucidated GB’s regulatory effects on antioxidant enzyme systems, specifically focusing on peroxidase (POD), superoxide dismutase (SOD), ascorbate peroxidase (APX), and catalase (CAT) activities. These findings will provide a comprehensive theoretical framework and practical methodology for maintaining jujube fruit quality under refrigeration conditions.

## 2. Material and Methods

### 2.1. Jujube Samples and Treatments

Hupingzao jujube fruit was sampled with light green skin color (white maturity) on 30 August 2022, from a jujube garden in Taigu, Jinzhong, Shanxi Province, China. Postharvest jujube fruits were placed in polyethylene bags and immediately transported to the laboratory. The jujube with a uniform fruit stalk, without mechanical damage or disease, was selected for experiments. Jujubes with fruit stalks were selected for the experiments. These jujubes were uniform in appearance, and free from mechanical damage and diseases. These jujube samples were pre-cooled at 4 °C for 24 h, then randomly assigned into two groups: (1) glycine betaine (GB) treated group: this fruit was dipped in 15 mmol/L GB solution for 10 min (the concentration and soak time obtained by preliminary experiment); (2) the control group: this fruit was dipped in distilled water for 10 min. After being naturally air dried in the laboratory, the two groups were, respectively, placed in polyethylene bags containing holes (0.03 mm) and stored at 0 ± 1 °C for 100 days (85–90% relative humidity). Each group had 540 jujube fruits. The fruit samples of each group were taken out to evaluate the physicochemical quality after being cold-stored 0, 20, 40, 60, 80, and 100 d, respectively, and three replicates were performed. At the same time, the sampled jujube fruits were cut into small pieces and rapidly frozen in liquid nitrogen, then stored at −80 °C in a freezer.

### 2.2. Firmness, Respiration Rate, and Weight Loss Assessments

The fruit firmness, the respiration rate, and weight loss were measured on the basis of Shi et al., method [6], respectively. The firmness was determined by a texture analyzer (TMS-PRO, Food Technology Corporation, Columbia, SC, USA); about 500 g of fruit from each group was placed in a 1 L preservation box with a sealed cover and kept for 2 h, then the respiration rate (CO_2_ mg kg^−1^ h^−1^) was determined by a digital respiration analyzer meter (FK-GH10, Fangke Instrument Co., Ltd., Shandong, China); thirty fruits of each group were selected to measure weight loss, expressed as weight loss rate (%).

### 2.3. Color Assessment

From each group, 15 jujube fruits were applied to measure the fruit color, which was quantified in L*, a*, and b* values using a chroma meter. The color values were obtained by four spots of each jujube fruit and averaged.

### 2.4. Total Soluble Solids and Titratable Acid Assessments

The pulp juice of 15 jujube fruits was applied to assay the total soluble solids (TSS) and titratable acid (TA). The TSS content was measured by a portable refractometer (WZB 45, Jingke, Shanghai, China), and the TA content was determined according to the acid–base neutralization titration method and expressed as the percentage of malic acid [17].

### 2.5. The Content of Antioxidant (Ascorbic Acid, Total Flavonoids, Total Phenolics, and Proanthocyanidins) Assessments

The pulp (10 g) from 15 jujube fruits was used for measuring the ascorbic acid (AsA) content according to the method of Sang et al. [18], expressed as g kg^−1^.

The contents of total phenolics and total flavonoids were determined by the method of Fan et al. [19]. The pulp (4.0 g) from 15 jujube fruits of each group was weighted, mixed with 40 mL of 80% (*v*/*v*) ethanol, then ground to a homogenate and transferred to a centrifuge tube, followed by ultrasonic extraction (20 min) and centrifuging at 10,000× *g* (10 min); three replicates were performed. These supernatants were collected and then concentrated to 15 mL by a rotary evaporator at 40 °C. The extracting solution (1.0 mL) was mixed with Folin–Ciocalteu reagent (0.5 mL) and incubated for 6 min, then 10% (*w*/*w*) Na_2_CO_3_ (1.0 mL) and deionized water (7.5 mL) were added and shaken. The reaction liquid was put in the dark (50 min), then measured at 760 nm. The total phenolic content was expressed as g kg^−1^.

For determining the total flavonoid content (g kg^−1^), the extracting solution (2.0 mL) was mixed with 5% (*w*/*w*) NaNO_2_ (2.0 mL); after reacting for 5 min, 10% (*w*/*w*) AlCl_3_ solution (0.15 mL) was added, and after reacting for another 5 min 1.0 mol L^−1^ NaOH (4.0 mL) and deionized water (1.85 mL) were added. After reacting for 5 min in the dark, it was then measured at 510 nm.

For determining the proanthocyanidins content (g kg^−1^), the extracting solution (1.0 mL) was mixed with 40 g L^−1^ vanillin methanol solution (6.0 mL) and concentrated hydrochloric acid (3.0 mL); the mixed liquid was then put in the dark for 900 min, then measured at 500 nm.

### 2.6. The Malondialdehyde (MDA) Content, Hydrogen Peroxide Content, and the Inhibition Ability of Superoxide Anion Production Assessments

The pulp (2 g) from five jujube fruits was applied to assess the MDA content (umol kg^−1^) based on the thiobarbituric acid (TBA) colorimetry method [20].

Hydrogen peroxide (H_2_O_2_) can react with molybdic acid, resulting in the formation of a complex. The quantity of H_2_O_2_ produced can be determined by measuring its absorbance at 405 nm. The content of H_2_O_2_ in jujube fruit was determined by spectrophotometry method using an assay kit (Jianchengkeji, Nanjing, China) and expressed as mmol g^−1^ protein.

The inhibition ability of the superoxide anion (O_2_^−^) was determined by the colorimetry method using an assay kit (Jianchengkeji, Nanjing, China) and expressed as U g^−1^ protein. The inhibition ability of O_2_^−^ by each gram of tissue is equivalent to the value of the inhibition ability of O_2_^−^ by 1 mg vitamin C, representing one activity unit.

### 2.7. The Activity of Antioxidant Enzymes Assessments

The activities of peroxidase (POD), superoxide dismutase (SOD), catalase (CAT), and ascorbate peroxidase (APX) in jujube fruit were measured by a POD, SOD, CAT, and APX assay kit (Jianchengkeji, Nanjing, China), respectively, and handled according to the instructions of the manufacturer.

POD activity was determined spectrophotometrically by monitoring the oxidation of H_2_O_2_ at 420 nm, based on the principle of the POD-catalyzed reaction. SOD exhibits a specific inhibitory effect on O_2_^−^, thereby reducing nitrite formation. During colorimetric analysis (550 nm), the absorbance value of the sample tube is consistently lower than that of the control tube. SOD activity in the tested sample is subsequently calculated. CAT can decompose H_2_O_2_; the reaction was terminated by adding ammonium molybdate, which reacted with residual H_2_O_2_ to form a light-yellow complex. The absorbance change at 405 nm was measured, and CAT activity was calculated accordingly. APX activity was quantified by monitoring the oxidation of ASA to monodehydroascorbic acid in the presence of H_2_O_2_. The reaction-induced decrease in absorbance at 290 nm was measured spectrophotometrically, and APX activity was calculated based on the decrease rate at 290 nm per unit time.

### 2.8. Statistical Analysis

Data were subjected to one-way analysis of variance (ANOVA) using SPSS 19.0 (SPSS Inc., Chicago, IL, USA). The results were expressed as the means ± standard error (SE). Significant differences (* *p* < 0.05 or ** *p* < 0.01) between the treatment group and control group were identified through Duncan’s multiple range test, with statistical significance thresholds indicated accordingly.

## 3. Results and Discussion

### 3.1. The Roles of GB Treatment on the Jujube Firmness, Respiration Rate, and Weight Loss Rate

**Figure 1:**
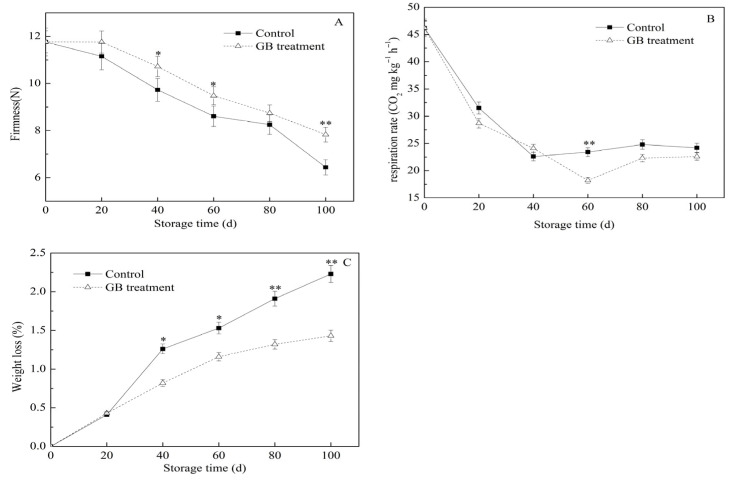
The effects of GB treatment on firmness (**A**), respiration rate (**B**), and weight loss (**C**) of Hupingzao jujube fruit during cold storage. Error bars represent the standard deviations of triplicate replications ± SE. * and ** indicate significant differences between GB-treated and control fruit *p* < 0.05 and *p* < 0.01, separately.

Fruits continue to undergo respiration, ripening, and senescence processes even after being harvested. The respiration rate significantly influences the fruit quality and storage duration [21]. In the case of jujube fruits, senescence is typically characterized by a reduction in firmness, weight loss, and the occurrence of rotting [22]. The weight loss in jujube fruits primarily results from water loss, which is commonly caused by respiration and evaporation, and this can have an impact on the economic value of the fruits. Firmness is also a crucial determinant when assessing the quality and consumer acceptability of fruits [23]. During storage, the firmness and weight of postharvest Hupingzao jujube fruits gradually decline [6]. In the study, as shown in Figure 1A, the firmness of jujube fruits, regardless of whether they were treated with GB or not, showed a progressive decrease during storage under low-temperature conditions. However, the reduction in firmness was less pronounced in the GB-treated fruits compared to the control group. On the 40th, 60th, and 100th days of cold storage, the firmness values of GB-treated fruits were 10.72 N, 9.48 N, and 7.83 N, respectively, which were significantly higher than those of the control fruits (* *p* < 0.05; ** *p* < 0.01). During cold storage, the respiration rate of jujube fruits initially declined and then increased (Figure 1B). From the 60th to the 100th day, the respiration rate in the GB treatment group was lower than that of the control group. As shown in Figure 1C, the weight loss of jujube fruits in both groups increased with the extension of cold storage time. On the 40th, 60th, 80th, and 100th days, the weight losses in the GB treatment group were only 0.82%, 1.16%, 1.32%, and 1.43%, respectively, which were significantly lower (* *p* < 0.05; ** *p* < 0.01) than those in the control group. These results indicated that GB treatment could inhibit the decrease in firmness, delay the increase in respiration rate, and significantly reduce weight loss in jujube fruits. Similarly to our findings, previous research has reported that GB treatment can inhibit the decline in firmness and weight loss in winter jujubes [3]. Additionally, it has been shown that treating pears with 5 mmol L^−1^ GB can retard the respiration rate and weight loss, while maintaining fruit firmness [24].

### 3.2. The Role of GB Treatment on Jujube Fruit Peel Color

**Figure 2:**
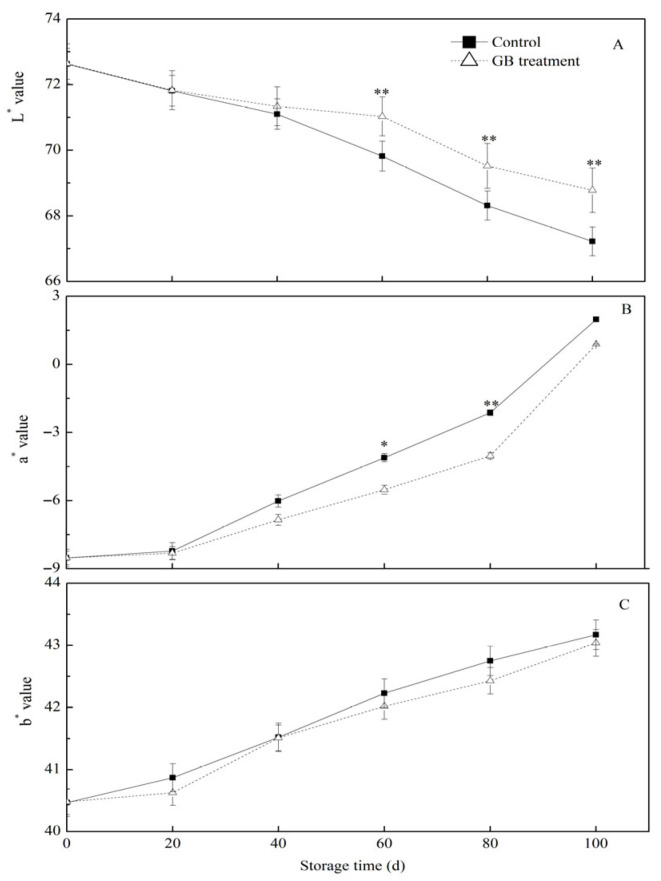
The effects of GB treatment on the peel color change, L* (**A**), a* (**B**), and b* (**C**), of Hupingzao jujube fruit during cold storage. Error bars represent the standard deviations of triplicate replications ±SE. * and ** indicate significant differences between GB-treated and control fruit *p* < 0.05 and *p* < 0.01, separately.

The peel color is a crucial characteristic influencing the consumer acceptability of fruits. Jujube fruit is particularly prone to color change, especially turning red during storage [25,26,27]. Thus, this study monitored the changes in the peel color of jujube fruits during cold storage. Typically, the brightness of a fruit peel is denoted by the L* value; the color transitions from green to red and blue to yellow are represented by a* and b* values, respectively [28]. As shown in Figure 2A, the luminance of jujube fruit peels in both groups gradually decreased over the storage period. After 60 days of storage, the luminance of the peel of GB-treated jujubes was significantly higher (** *p* < 0.01) compared to the control group. As the storage time extended, the fruit peel color gradually shifted towards yellow, and the redness changed from partial to fully red. Consequently, the values of a* and b* exhibited a gradual upward trend (Figure 2B,C). In the later stage of storage, the degree of redness of the fruit peel in the GB treatment group was significantly lower than that of the control group (Figure 2B). However, there was no significant difference in the degree of yellowing of the jujube pericarp between the GB-treated and the control groups. These results suggested that an exogenous GB treatment could preserve the luminance of the pericarp and reduce the degree of peel redness in cold-stored jujube fruits. Previous research has also demonstrated that GB treatment could effectively mitigate the browning of pears and peaches [14,29].

### 3.3. The Roles of GB Treatment on Jujube Fruit TSS and TA Content

**Figure 3:**
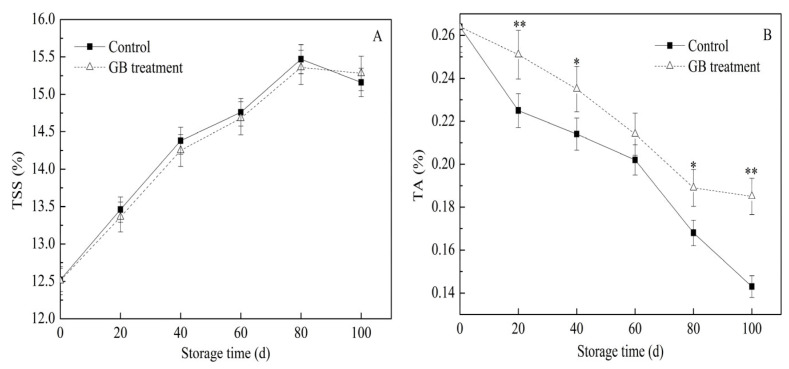
The effects of GB treatment on TSS content (**A**) and TA content (**B**) of Hupingzao jujube fruit during cold storage. Error bars represent the standard deviations of triplicate replications ±SE. * and ** indicate significant differences between GB-treated and control fruit *p* < 0.05 and *p* < 0.01, separately.

TSS is a key indicator for evaluating the quality of jujube fruits, and the TSS content increases during the ripening process [22,30]. Nitric oxide (NO) treatment has been reported to slow down the increase in TSS content in jujubes [27]. As shown in Figure 3A, the TSS contents in both the control group and the GB-treated jujube fruits initially increased and then decreased. The peak values of TSS content, which were 15.47% in the control group and 15.36% in the GB-treated group, were both reached on the 80th day of storage during the storage period. This phenomenon might be attributed to the consumption of sugars and other substances in the fruits during the later stage of storage. The results were consistent with those of a previous study on winter jujubes treated with GB [3].

TA is another crucial parameter for assessing the flavor and nutritional quality of jujube fruit. Generally, the TA content decreases as the fruit ripens [31]. In this study, the TA contents in both groups of jujube fruits showed a downward trend during storage (Figure 3B). With the exception of the 60th day, the TA content in the GB-treated fruits was significantly higher (* *p* < 0.05, ** *p* < 0.01) than that of the control fruits. Notably, from the 80th to the 100th day, the TA content in GB-treated fruits decreased by only 2.1%. These results were in line with the trends reported in previous studies [27,30]. They indicate that an exogenous GB treatment can significantly slow down the decline of TA in cold-stored jujube fruits, which helps to maintain the flavor and nutritional quality of the fruits.

### 3.4. The Roles of GB Treatment on Jujube Fruit Antioxidants Content

**Figure 4:**
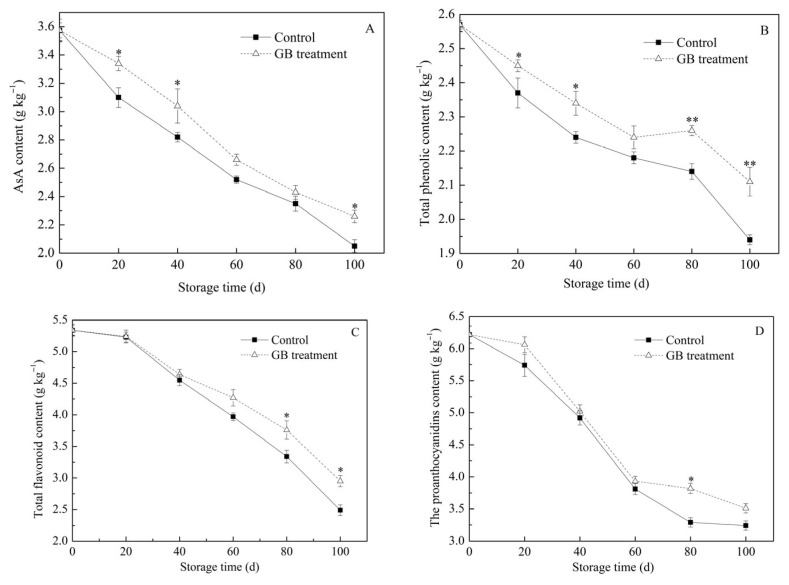
The effects of GB treatment on AsA content (**A**), total phenolic content (**B**), total flavonoid content (**C**), and proanthocyanidins content (**D**) of Hupingzao jujube fruit during cold storage. Error bars represent the standard deviations of triplicate replications ±SE. * and ** indicate significant differences between GB-treated and control fruit *p* < 0.05 and *p* < 0.01, separately.

The content changes in antioxidants, including ascorbic acid (AsA), polyphenols, flavonoids, and proanthocyanidins, in the two groups of jujube fruits are presented in Figure 4A–D. AsA is not only a vital nutrient but also a significant antioxidant for scavenging ROS in fruits [32]. It can directly eliminate ROS and serves as a substrate for APX to catalyze the decomposition of H_2_O_2_ [33]. However, the AsA content in postharvest jujube fruits typically decreases continuously during storage [26]. In the study, the AsA content in both groups of jujube fruits gradually declined during cold storage (Figure 4A). On the 20th, 40th, and 100th days of storage, the AsA contents in GB-treated fruits were 3.34 g kg^−1^, 3.04 g kg^−1^, and 2.26 g kg^−1^ respectively, which were significantly higher (* *p* < 0.05) than those in the control group. These results suggest that an exogenous GB treatment can effectively inhibit the decline of AsA content in cold-stored jujube fruits. Previous studies have reported that treatments such as chitosan/nano-silica/sodium alginate coating and 1-MCP can inhibit the oxidative degradation of AsA in jujube fruits [20,26]. Additionally, Chen et al. [15] found that GB treatment could increase the AsA content in bananas and enhance their cold resistance during cold storage. This phenomenon might be attributed to the fact that GB treatment can reduce the enzymatic reaction rate and the respiration rate of fruits, thereby slowing down the oxidation and decomposition of AsA [19].

The flavonoids, phenols, and proanthocyanidins are essential secondary metabolites in fruits. They can enhance the oxidation resistance of fruits. Jujube fruits are rich in these substances, which play a vital role in scavenging free radicals and preventing excessive ROS accumulation [21,27,33]. Previous studies have indicated that the content of these antioxidants varies with fruit maturity, storage duration, and postharvest treatments [34]. As depicted in Figure 4B, the total phenolic content in both groups of jujube fruits generally decreased during storage. With the exception of the 60th day, the total phenolic content in the GB-treated group was significantly higher than that of the control group (* *p* < 0.05; ** *p* < 0.01). As shown in Figure 4C,D, the contents of total flavonoids and proanthocyanidins in both groups of fruits continuously declined during cold storage. And the contents in the GB-treated fruits were higher than those in the control fruits. On the 100th day, the total flavonoid content in the GB-treated fruits was 2.95 g kg^−1^, while that of the control group was 2.49 g kg^−1^, which was significantly lower (* *p* < 0.05) compared to the GB-treated group (Figure 4C). On the 80th day, the proanthocyanidin content in the GB-treated fruits was 3.82 g kg^−1^, which was significantly higher than that of the control group (Figure 4D).

These results may be attributed to the fact that the overall antioxidant capacity of the fruits gradually weakens as the storage time prolongs, leading to the oxidation and decomposition of antioxidant substances [35]. The application of an exogenous GB treatment can maintain the content of antioxidants in cold-stored jujube fruits, suggesting that GB treatment is an effective approach to enhance the antioxidant capacity of fruits at low temperatures. Previous studies have reported that the contents of total phenolics and total flavonoids in jujube fruits decrease during cold storage, but exogenous substance treatments can increase these contents [20,26]. Moreover, GB treatment has been shown to effectively maintain higher levels of antioxidants in cold-stored bananas [15].

### 3.5. The Roles of GB Treatment on Jujube Fruit MDA Content, H_2_O_2_ Content and the Inhibition Ability of O_2_^−^

**Figure 5:**
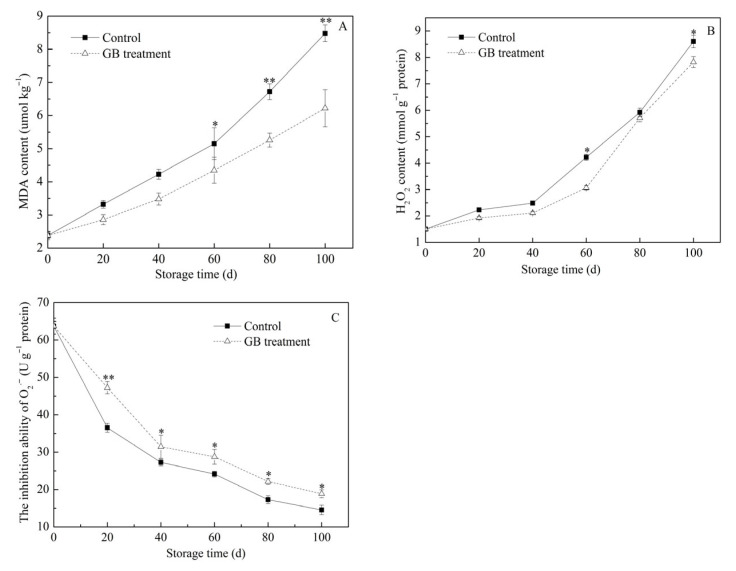
The effects of GB treatment on MDA content (**A**), H_2_O_2_ content (**B**), and the inhibition ability of O_2_^−^ (**C**) in Hupingzao jujube fruit during cold storage. Error bars represent the standard deviations of triplicate replications ±SE. * and ** indicate significant differences between GB-treated and control fruit *p* < 0.05 and *p* < 0.01, separately.

Membrane lipid peroxidation can cause the deterioration of postharvest fruit quality, and the accumulation of ROS exacerbates the degree of peroxidation. ROS encompasses O_2_^−^, ·OH, H_2_O_2_, and ·(ROOH) [36]. The accumulation of H_2_O_2_ can lead to the loss of membrane integrity and a reduction in fruit quality [37]. MDA is a marker of cellular membrane lipid peroxidation, and its content is used as an indicator of the degree of cell membrane damage [27,38]. In the study, the MDA and H_2_O_2_ content in jujube fruits from both groups increased as the refrigeration time extended (Figure 5A,B). Throughout the entire storage period, the MDA and H_2_O_2_ contents in the GB-treated fruits were lower. In particular, on the 100th day, the MDA content in the GB-treated fruit was only 6.22 umol kg^−1^, which was significantly lower than that of the control (8.48 umol kg^−1^). On the same day, the H_2_O_2_ content in the GB-treated fruits decreased by 9.04% compared to the control. It has been reported that low MDA and H_2_O_2_ contents are associated with antioxidant enzyme activities [12]. As shown in Figure 5C, the ability to inhibit O_2_^−^ continuously decreased during the entire storage time. However, the inhibition ability of the GB-treated fruit was significantly higher than that of the control group. These results showed that the GB treatment could prevent the increase in MDA and H_2_O_2_ contents and enhance the ability to inhibit O_2_^−^. The findings of this study are consistent with previous reports showing that GB treatment can reduce the MDA and ROS content in blood oranges, pears, and winter jujubes during cold storage [3,14,39].

### 3.6. The Roles of GB Treatment on Jujube Fruit Antioxidant Enzyme Activities

**Figure 6:**
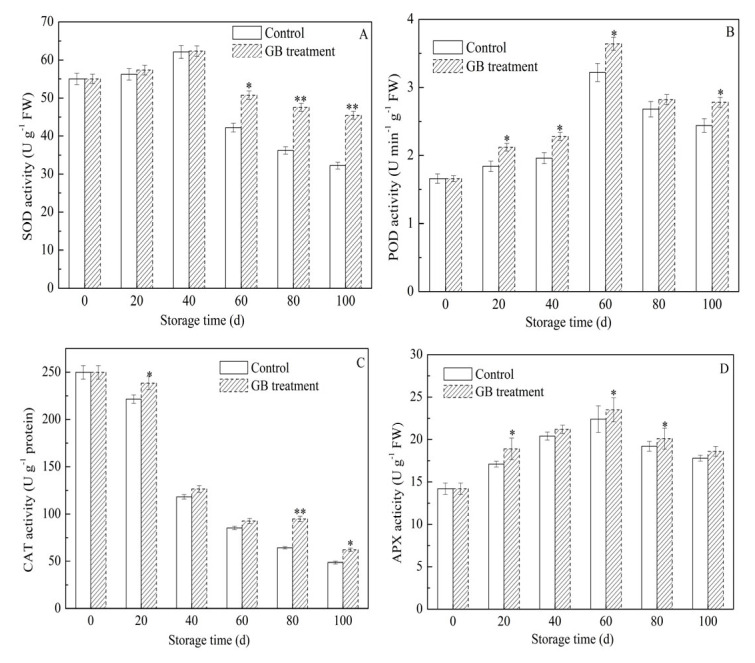
The effects of GB treatment on antioxidant enzyme activities of SOD (**A**), POD (**B**), CAT (**C**), and APX (**D**) in Hupingzao jujube fruit during cold storage. Error bars represent the standard deviations of triplicate replications ±SE. * and ** indicate significant differences between GB-treated and control fruit *p* < 0.05 and *p* < 0.01, separately.

CAT, APX, SOD, and POD are key antioxidant enzymes within the cellular defense system, which play a crucial role in eliminating ROS [35]. Specifically, CAT, APX, and POD can catalyze the decomposition of H_2_O_2_ into H_2_O and O_2_, thereby protecting fruits from the toxicity of H_2_O_2_ and delaying fruit senescence [31,40]. SOD serves as the primary line of defense in the cell’s natural response against oxidative stress. It scavenges O_2_^−^, singlet oxygen, and inhibits the formation of hydroxyl radicals [41]. Consequently, elevated activities of these antioxidant enzymes can impede the accumulation of ROS and preserve fruit quality [15,42]. Nevertheless, the antioxidant capacity diminishes when fruits are exposed to low temperatures for extended periods [12]. The impacts of GB treatment on the activities of SOD, POD, APX, and CAT in cold-stored jujube fruits are presented in Figure 6A–D. Throughout the storage period, the activities of SOD, POD, and APX in both groups initially increased and then decreased. In contrast, the CAT activity continuously declined. Moreover, the activities of antioxidant enzymes in jujube fruits treated with GB were higher. On the 20th and 40th days of storage, the SOD activity in GB-treated jujube fruits was nearly at the same level as that of the control group. However, on the 60th, 80th, and 100th days, the SOD activity in GB-treated fruits was 20.20%, 31.13%, and 41.06% higher, respectively, than that of the control fruits, and these differences were statistically significant (Figure 6A). Similarly, on the 20th, 40th, 60th, and 100th days, the POD activity in GB-treated fruits was significantly higher (* *p* < 0.05) than that of the control fruit (Figure 6B). As depicted in Figure 6C, on the 20th, 80th, and 100th days, the CAT activity in GB-treated fruits was 8.50%, 47.28%, and 27.72% higher, respectively, than that of the control fruits, with these differences being statistically significant (* *p* < 0.05, ** *p* < 0.01). On the 20th, 60th, and 80th days of storage, the APX activity in jujube fruits treated with GB was 4.91%, 4.69%, and 4.49% higher, respectively, than that of the control group, and these differences were statistically significant (Figure 6D). Given the above, these findings indicated that GB treatment could enhance the antioxidant capacity of cold-stored jujube fruits by up-regulating the activities of antioxidant enzymes, thereby contributing to maintaining the balance of ROS and delaying the postharvest senescence of jujube fruit. Consistent with our experimental results, the positive effects of GB treatment on increasing the activities of SOD, POD, APX, and CAT have also been reported in various fruits, including zucchini [42], pears [14], bananas [15], blood oranges [40], and winter jujubes [3].

## 4. Conclusions

In conclusion, GB treatment demonstrated comprehensive preservation effects on the quality of postharvest Hupingzao jujubes during cold storage. GB treatment significantly delayed the fruit softening progression, suppressed the respiration metabolism, mitigated weight loss, and induced a reduction in peel redness. And GB treatment promoted the increase in antioxidant substance content (AsA, total phenols, total flavonoids, proanthocyanidins), reduced the accumulation of MDA and H_2_O_2_ contents, increased the inhibition ability of O_2_^−^, and enhanced the activities of antioxidant enzymes (SOD, POD, APX, CAT). In summary, GB treatment represents a novel and effective approach to maintaining postharvest quality in Hupingzao jujubes during cold storage by enhancing the antioxidant system of the jujube fruit. These findings demonstrated that GB treatment could be a promising postharvest strategy for enhancing the quality of postharvest fruits during storage.

## Data Availability

The data presented in this study are available on request from the corresponding author. The data are not publicly available due to avoiding the data being analyzed and published by others beforehand.
